# Impact of GAN-based lesion-focused medical image super-resolution on the robustness of radiomic features

**DOI:** 10.1038/s41598-021-00898-z

**Published:** 2021-11-01

**Authors:** Erick Costa de Farias, Christian di Noia, Changhee Han, Evis Sala, Mauro Castelli, Leonardo Rundo

**Affiliations:** 1 NOVA Information Management School (NOVA IMS), Universidade Nova de Lisboa, 1070-312 Lisbon, Portugal; 2 Department of Physics, University of Milano-Bicocca, 20126 Milan, Italy; 3 Saitama Prefectural University, Saitama, 343-8540 Japan; 4 Department of Radiology, University of Cambridge, Cambridge, CB2 0QQ UK; 5 Cancer Research UK Cambridge Centre, University of Cambridge, Cambridge, CB2 0RE UK

**Keywords:** Biomarkers, Cancer, Medical imaging, Biomarkers, Cancer imaging, Lung cancer, Biomedical engineering

## Abstract

Robust machine learning models based on radiomic features might allow for accurate diagnosis, prognosis, and medical decision-making. Unfortunately, the lack of standardized radiomic feature extraction has hampered their clinical use. Since the radiomic features tend to be affected by low voxel statistics in regions of interest, increasing the sample size would improve their robustness in clinical studies. Therefore, we propose a Generative Adversarial Network (GAN)-based lesion-focused framework for Computed Tomography (CT) image Super-Resolution (SR); for the lesion (i.e., cancer) patch-focused training, we incorporate Spatial Pyramid Pooling (SPP) into GAN-Constrained by the Identical, Residual, and Cycle Learning Ensemble (GAN-CIRCLE). At $2\times$ SR, the proposed model achieved better perceptual quality with less blurring than the other considered state-of-the-art SR methods, while producing comparable results at $4\times$ SR. We also evaluated the robustness of our model’s radiomic feature in terms of quantization on a different lung cancer CT dataset using Principal Component Analysis (PCA). Intriguingly, the most important radiomic features in our PCA-based analysis were the most robust features extracted on the GAN-super-resolved images. These achievements pave the way for the application of GAN-based image Super-Resolution techniques for studies of radiomics for robust biomarker discovery.

## Introduction

Recently, medical image analysis has been revolutionized by available large-scale datasets and technology advancements in statistics and artificial intelligence. In particular, combining radiomics^1^—an approach to extract quantitative features from medical images—and machine learning has obtained meaningful clinical insights. Robust machine learning models based on large-scale radiomic features might allow for accurate diagnosis, prognosis, and medical decision-making; of course, thoroughly considering the whole radiomic processes is essential to obtain these reliable models.

Despite the potential of radiomics, high quantitative feature variability across different software implementations has hampered its clinical use^2,3^. This phenomenon derives from the lack of standardized definitions and extraction of radiomic features with validated reference values. To tackle this limitation and facilitate clinical interpretation, the Image Biomarker Standardization Initiative^2^ produced and validated the reference values for commonly-used radiomic features. However, as the paper’s authors highlighted, image features still need to be robust against differences in acquisition, reconstruction, and segmentation to ensure reproducibility. For this reason, recent studies have investigated the robustness of radiomic features in several scenarios and applications using heterogeneous datasets. Several sources of variability have been assessed, such as image and region of interest (ROI) perturbations^4,5^, slice thickness variations^6,7^, and different resampling strategies^8^. Since the radiomic features might tend to be affected by low statistics in ROI voxels, we hypothesize that increasing such a sample size would increase the robustness of radiomic features in clinical studies. Therefore, we aim to apply image Super-Resolution (SR) to increase the number of voxels used in the computation of radiomic features.

Generative Adversarial Networks (GANs) have been commonly exploited for Data Augmentation (DA), along with image SR^9^, thanks to their ability to improve feature robustness. Sandfort et al.^10^ used CycleGAN^11^-based DA for Computed Tomography (CT) segmentation by translating contrast images into synthetic non-contrast ones. To maximize the DA effect with GAN combinations, Han et al.^12^ proposed a two-step GAN-based DA approach that generates and refines brain Magnetic Resonance (MR) images with/without tumors separately. Considering the GAN-based DA’s interpolation/extrapolation effect, GAN may remarkably help achieve reference values for radiomic features. The most prominent work on CT image SR is GAN Constrained by the Identical, Residual, and Cycle Learning Ensemble (GAN-CIRCLE)^13^, outperforming previous works^14–17^. GAN-CIRCLE can preserve anatomical information and suppress noise, leading to excellent diagnostic performance in terms of traditional image quality metrics^13,18^. For example, Guha et al.^18^ exploited GAN-CIRCLE to super-resolve trabecular bone microstructures and improved the structural similarity index. Meanwhile, GAN-based lesion-focused medical image SR can improve SR performance around lesions, especially for downstream radiomic analyses^19^. Along with GAN-based medical image SR, novel approaches based on progressive GANs^20^ and attention mechanisms^21^ have been recently applied to video SR.

For the first time, in this paper, we evaluate the robustness of radiomic features extracted from super-resolved images by GAN-SR and bicubic interpolation. The authors incorporated Spatial Pyramid Pooling (SPP)^22^ into the discriminator of GAN-CIRCLE^13^ to handle different input CT image sizes for patch-focused training in lesions; we cropped the input CT images to their lesion bounding boxes to reduce training costs and improve image quality (e.g., fewer artifacts)^19^. Along with perceptual quality evaluation, we also assessed the robustness of radiomics, in terms of quantization, for our model against a bicubic interpolation baseline on a separate lung cancer CT dataset. We found that the most important radiomic features in our Principal Component Analysis (PCA)-based examination were the most robust features extracted on the GAN-super-resolved images.

To summarize, this work provides the following contributions:

- definition of the first GAN-based, lesion-focused, SR framework for CT images;
- comparison with state-of-the-art SR techniques highlighting the suitability of the proposed framework;
- at $2\times$ SR, the images are characterized by better perceptual quality, as suggested by the peak signal-to-noise ratio and structural similarity index measures, on a large-scale dataset;
- at $4\times$ SR, the proposed GAN-based model achieves comparable results to the ones obtained by state-of-the-art SR techniques;
- the proposed GAN-SR framework improves the robustness of the most important radiomic features in an independent lung CT dataset.

## Materials and methods

### Analyzed CT datasets

#### DeepLesion dataset

As a subset of the DeepLesion dataset^23^, which contains 10, 594 scans of 4, 427 patients, our study exploits 10, 000 CT slices with an image size of $512 \times 512$ pixels and in-plane pixel spacing between 0.18 and 0.98 mm (median: 0.82 mm). The dataset contains diverse lesion images for various body parts with 2D lesion information on diameter measurements, bounding boxes, and semantic labels. We use the DeepLesion dataset to train a GAN-CIRCLE model for SR.

#### NSCLC-radiomics dataset

The Non-Small Cell Lung Cancer-Radiomics (NSCLC-Radiomics) dataset^24^ is a well-established publicly available dataset that contains CT slices from 422 NSCLC patients. For careful and reliable radiomic analyses, our study uses a highly homogeneous subset composed of 142 CT scans, accounting for 17, 938 CT slices with an image size of $512 \times 512$ pixels, in-plane pixel spacing of 0.98 mm, and slice thickness of 3.00 mm. The B19f convolution kernel was applied on all the scans for CT image reconstruction.

The dataset provides annotated 3D tumor segmentation masks and clinical outcome data. The images are used to assess our proposed lesion-focused CIRCLE-GAN framework in terms of radiomic feature robustness.

### The proposed GAN-powered framework for radiomic feature robustness

#### Pre-processing

For all the implemented SR approaches, the range of intensity for raw CT volumes was clipped to $[-100, 400]$ Hounsfield Units (HU), and then normalized to [0, 1]. We generated the Low-Resolution CT (LRCT) counterparts from the High-Resolution CT (HRCT) images by degrading them through a Gaussian white noise process with a standard deviation of 0.25 and a Gaussian blur, with a kernel size of $8\times 8$ pixels and a bandwidth of 1.6. Afterwards, the images were downsampled with a scale of 2 and upsampled using the nearest neighbor interpolation, according to You et al.^13^. The upsampling step improves feature extraction by enforcing the same image size for LRCT and HRCT^25^. As in the original GAN-CIRCLE^13^, for convenience in the training of our proposed network, we upsampled the LR image *via* proximal interpolation to ensure that input and output have the same size. Image patches were then cropped based on the lesion bounding box annotations in the metadata—the cropping process leads to avoiding artifact generation out of the lesion area^19^. The preprocessing pipeline is displayed in Fig. 1.

By applying this procedure only on the Deeplesion dataset, we generated 10, 000 LRCT/HRCT patches with similar image sizes for training a CIRCLE-GAN-based SR model.

#### CIRCLE-GAN-based image super resolution

**Figure 1:**
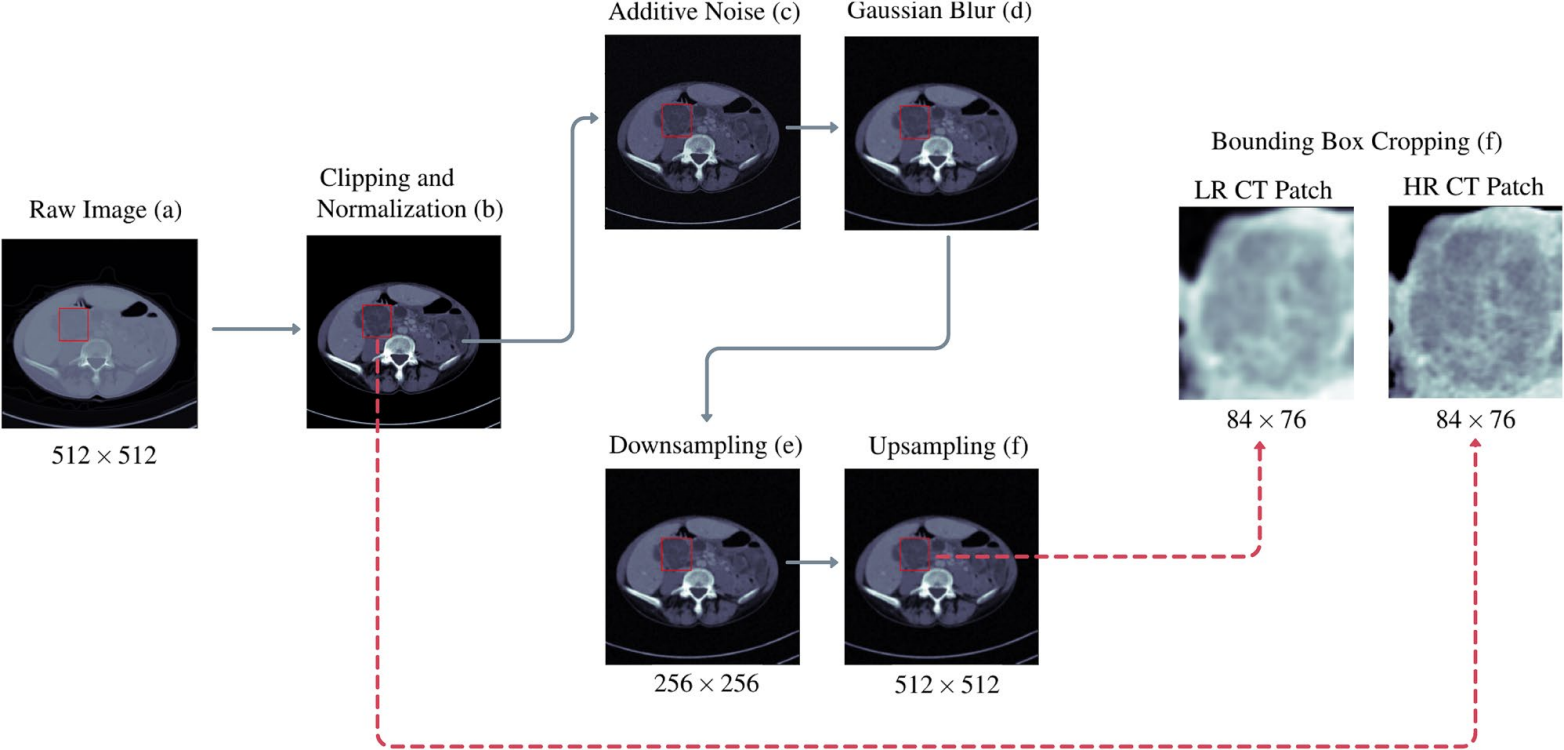
CT image preprocessing pipeline for GAN training. The HU values of input CT images (**a**) were clipped to the range $[-100, 400]$ HU and normalized to the unit range [0, 1] (**b**). To generate the low resolution CT image counterpart, the image was perturbed by noise addition (**c**) and Gaussian blurring (**d**), downsampled by a factor of $2\times$ (**e**) and then upsampled to the original dimension (**f**) using a nearest neighbor interpolation method. Finally, the HRCT patch and LRCT patch were extracted from the lesion bounding box crops (**g**).

##### Network architecture

 We used a modified version of CIRCLE-GAN^13^ to tackle the SR problem effectively. The CIRCLE-GAN is a cycle-consistent adversarial model consisting of two non-linear generative mappings and their respective discriminators that are trained jointly for optimal convergence.

The first generative mapping $G:\text {LR} \rightarrow \text {HR}$ attempts to generate a realistic high-resolution image ${\mathbf {I}}_\text {hr}$ that a discriminator $D_\text {HR}$ cannot distinguish from the real one, whereas a generative mapping $F: \text {HR} \rightarrow \text {LR}$ is responsible for generating a realistic low-resolution image ${\mathbf {I}}_\text {lr}$, not distinguishable by a discriminator $D_\text {LR}$. This minimax game is formulated as follows:

1 $$\begin{aligned} \min \limits _{G, F} \max \limits _{D_\text {HR}, D_\text {LR}} {\mathscr {L}}_\text {GAN}(G, D_\text {HR}) + {\mathscr {L}}_\text {GAN}(F, D_\text {LR}). \end{aligned}$$

The generator networks *G* and *F* share the same architecture, which consists of networks for feature extraction and reconstruction. The *feature extraction network* consists of twelve layers (i.e., feature blocks) of $3\times 3$ convolution kernels, bias, Leaky Rectified Linear Unit (ReLU) activation, and dropout. Each block output is concatenated through skip connections before the reconstruction network to capture local/global image features. The number of output filters in each convolutional layer is set according to You et al.^13^. In the *reconstruction network*, two branches are stacked in a network-in-network fashion to increase non-linearity and potentially reduce the filter space dimension for faster computation. A transposed convolutional layer with $\text {stride} = 2$ is adopted for upsampling and the last convolutional layer combines all feature maps to produce the SR output.

**Figure 2:**
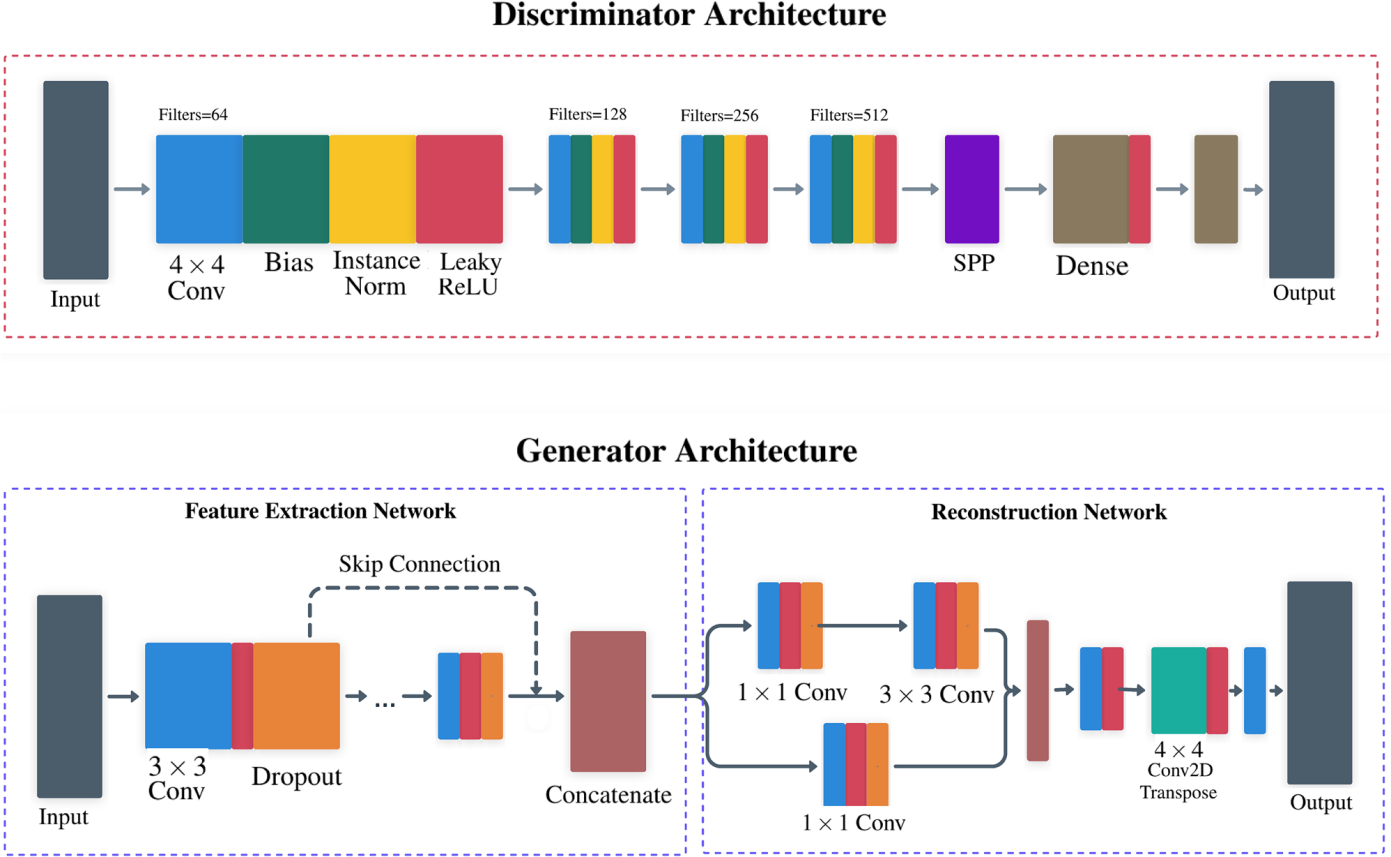
The discriminator and generator architectures devised for GAN-SR of medical images.

The discriminators $D_\text {HR}$ and $D_\text {LR}$ also share the same network architecture, which is composed of four blocks of $4\times 4$ convolution kernel, bias, instance normalization, and Leaky ReLU activation followed by an SPP layer and then two dense layers. Inspired by He et al.^26^, the SPP layer was added to handle multi-sized LRCT/HRCT input patches, allowing for the training of a lesion patch-focused network. Figure 2 displays the discriminator and generator architectures used in our work.

Similar to GAN-CIRCLE^13^, the loss function combines four different loss terms to regularize the training procedure by enforcing the desired mappings:

- an *adversarial loss term* (${\mathscr {L}}_\text {Adv}$) to enforce the matching of empirical distributions in the source and target domains;
- a $\ell _1$-norm *cycle-consistency loss term* (${\mathscr {L}}_\text {Cyc}$) to prevent degeneracy in the adversarial learning and promote forward and backward cycle consistency, defined as $G(F({\mathbf {I}}_\text {hr}) \approx {\mathbf {I}}_\text {hr}$ and $F(G({\mathbf {I}}_\text {lr})) \approx {\mathbf {I}}_\text {lr}$;
- a $\ell _1$-norm *identity loss term* (${\mathscr {L}}_\text {IDT}$) to regularize the training process and promote the relationships $G({\mathbf {I}}_\text {hr}) \approx {\mathbf {I}}_\text {hr}$ and $F({\mathbf {I}}_\text {lr}) \approx {\mathbf {I}}_\text {lr}$;
- a *joint sparsifying loss term* (${\mathscr {L}}_\text {JST}$) to promote image sparsity and reduced noise.

Thus, the overall loss function used for training is defined as:

2 $$\begin{aligned} {\mathscr {L}}_\text {CIRCLE} = {\mathscr {L}}_\text {Adv}(D_\text {HR}, G) + {\mathscr {L}}_\text {Adv}(D_\text {LR}, F) + \lambda _1 {\mathscr {L}}_{Cyc}(G, F) + \lambda _2 {\mathscr {L}}_\text {IDT}(G,F) + \lambda _3 {\mathscr {L}}_\text {JST}(G), \end{aligned}$$

where $\lambda _1$, $\lambda _2$ and $\lambda _3$ are weighting parameters to balance the different loss terms, respectively.

##### Implementation details

 The proposed network was trained in an end-to-end fashion to optimize the loss function; the convolution layers’ weights were initialized with a zero-mean Gaussian distribution, with a standard deviation of 2/*m*, where $m =f^2 \times n_f$, *f* is a filter size, and $n_f$ is the number of filters; this initialization can relieve diminishing gradients and improve the convergence of deep network architectures^27^.

The discriminators’ learning rate $\gamma _D$ was set to $10^{-5}$ equally for $D_\text {HR}$ and $D_\text {LR}$, while the learning rate for the generators *G* and *F* was set to $\gamma _G=\gamma _D/2$, following the Two Times Update Rule (TTUR)^28^, to improve GAN convergence under mild assumptions. Dropout regularization layers, applied in the generators, were initialized with the rate $p_\text {Dropout} = 0.8$. Leaky ReLU layers were initialized with the negative slope coefficient $\alpha = 0.1$. The loss weights $\lambda _1$, $\lambda _2$, and $\lambda _3$ were set to 1, 0.5 and 0.00001, respectively.

The training used the Adam optimizer with exponential decay rates of $\beta _1=0.5$ and $\beta _2=0.9$ during 100 epochs with batches of 16 images. On average, the training took 9-11 hours per iteration, using TensorFlow (version 2.3.0) on a shared HPC workspace with an Nvidia Tesla P100 Graphics Processing Unit (GPU). The implemented code is available under the GNU license on https://github.com/erickcfarias/SR-CIRCLE-GAN.

##### Model evaluation and comparisons

 To evaluate the trained model, conventional quantitative metrics—namely, Peak Signal-to-Noise Ratio (PSNR) and Structural Similarity Index Measure (SSIM)—were calculated on 1, 000 CT images held out for performance evaluation. As a baseline for comparison, we also resampled the images using a Bicubic interpolation method.

To test the effectiveness of our framework, we compared it with other state-of-the-art methods, namely: Image Super-Resolution Network with an Expectation-Maximization Attention Mechanism (EMASRN^21^), Enhanced Deep Super-Resolution (EDSR^29^), Cascading Residual Network (CARN^30^) and Super-Resolution based on Dictionary Learning and Sparse Representation (DLSR^31^). For the EMASRN model, we relied on the implementation available at https://github.com/xyzhu1/EMASRN, optimizing the network for $\ell _1$-norm loss during 1000 epochs with $T=4$, a batch size of 16, and a learning rate of $10^{-5}$ halved every 200 epochs. For the EDSR model, we trained the network with the Adam optimizer with $\beta _1 = 0.9$, $\beta _2 = 0.999$, optimizing for $\ell _1$-norm loss during 500 epochs, a batch size of 16, and a learning rate of $10^{-5}$ halved every 100 epochs. For the CARN model, we trained the network with the Adam optimizer with $\beta _1 = 0.9$, $\beta _2 = 0.999$, optimizing for $\ell _1$-norm loss during 500 epochs, a batch size of 16, and a learning rate of $10^{-5}$ halved every 100 epochs. For the DLSR model, we trained the dictionaries with a size of 2048 atoms, using 100, 000 randomly sampled patches, a sparsity regularization parameter $\lambda = 0.4$ and $5\times 5$-pixel patches with an overlap of 4 pixels between adjacent patches. We varied the upscale rate to generate the $2\times$ and $4\times$ versions for all the tested models.

To further assess the performance of the proposed GAN-CIRCLE-based SR method, at $4\times$ SR, we compared the native $4\times$ GAN-CIRCLE SR against the sequential application of two GAN-CIRCLE instances at $2\times$ SR, denoted as GAN-CIRCLE^x^.

#### Radiomic feature extraction

The radiomic features considered in this study were computed using PyRadiomics (version 2.2.0)^32^, an open-source Python package widely used for this purpose. Since this software requires image input to be in the Neuroimaging Informatics Technology Initiative (NIfTI) format^33^, a preliminary step was performed to convert the original Digital Imaging and Communications in Medicine (DICOM) scan and segmentation files to this format using custom software written in MATLAB (The Mathworks Inc., Natick, MA, USA) version R2019b.

Excluding the shape-based features and first-order features (since they are independent of the rebinning), 75 3D radiomic texture features were calculated without any image filters applied from the following categories: Gray-Level Co-occurrence Matrix features (GLCM)^34–36^ (24), Gray-Level Dependence Matrix (GLDM)^37^ (14), Gray-Level Run Length Matrix (GLRLM)^38^ (16), Gray-Level Size Zone Matrix (GLSZM)^39^ (16) and Neighboring Gray-Tone Difference Matrix Features (NGTDM)^40^ (5).

The radiomic features were extracted from the NSCLC radiomics CT dataset by using different quantization configurations: the number of bins varied in $\{8, 16, 32, 64, 128, 256\}$. By relying upon the slice thickness, which is the same for all CT scans included in this homogeneous subset of the whole NSCLC dataset, 3D feature computation without any resampling was used to avoid interpolation artifacts.

#### Radiomic feature robustness analysis

The intraclass correlation coefficient (ICC) was computed to identify which features are correlated with the number of bins used during the quantization step. Given *k* multiple measurements to be compared (i.e., 6 different rebinnings), $\text {ICC}(3,1)$^41^ for a two-way random-effects (or mixed effects) model was used:

3 $$\begin{aligned} \text {ICC}(3,1) = \frac{\text {MS}_R - \text {MS}_E}{\text {MS}_R + (k-1) \text {MS}_E}, \end{aligned}$$

where $\text {MS}_R$ and $\text {MS}_E$ are the mean square for rows and mean square for error, respectively.

According to the ICC values^42^, we divided the features into:

- Poor robustness: ICC $\le$ 0.5;
- Moderate robustness: 0.5 < ICC $\le$ 0.75;
- Good robustness: 0.75 < ICC $\le$ 0.9;
- Excellent robustness: ICC > 0.9.

We investigated how the robustness of the textural features (in terms of ICC) varies according to the different groups of images. For each group, with the aim of identifying the most robust features, the ICC was calculated by varying the number of bins considered $\{8, 16, 32, 64, 128, 256\}$. By doing so, we determined the number of robust features by varying the number of bins in the quantization step. After determining the features showing excellent robustness, we aimed to identify the most relevant features for the analysis at hand; for this purpose, we used in an agnostic way the most best known technique of dimensionality reduction: the PCA^43^. For this purpose, we had to select a specific quantization setting binning; therefore, the different number of bins were perturbed, *via* mathematical morphology operations, to select the most robust setting. With more details, the original ROIs were perturbed using morphological operators (opening and closing with a 3D spherical structuring element of 1-pixel radius). Accordingly, we produced three versions for each ROI (i.e., original, opening, and closing). This procedure simulates ROI variations through consideration of intra-/inter-reader dependence during manual contouring^44^. The optimal number of bins was selected after the ROI perturbation process, by considering the rebinning with the highest number of robust features. It is worth noting that the optimal binning was selected on the Original images and not on the super-resolved ones, thus adopting the most conservative choice for fair comparisons.

With the goal of carefully analyzing these variations in terms of ICC, and after the selection of the optimal rebinning setting, we assessed the importance of these features by means of a ranking procedure: we performed a PCA and we calculated a weighted average of the features extracted from the Original images, according to the first three Principal Components (PCs), to assess their relative importance. In particular, we calculated the correlation matrix (as well as the eigenvectors and eigenvalues of the correlation matrix) to identify the PCs. PCs represent the directions of the data that explain a maximum amount of variance, i.e., the directions that capture most of the relevant and non-redundant information in the data. Then, to determine the relative importance of the features for the PCs considered, we used a quadrature sum for the individual features related to the different PCs. In this way, we determined a ranking of the features by the study of their relative weights in the main components considered.

## Results

### Image super-resolution results

**Figure 3:**
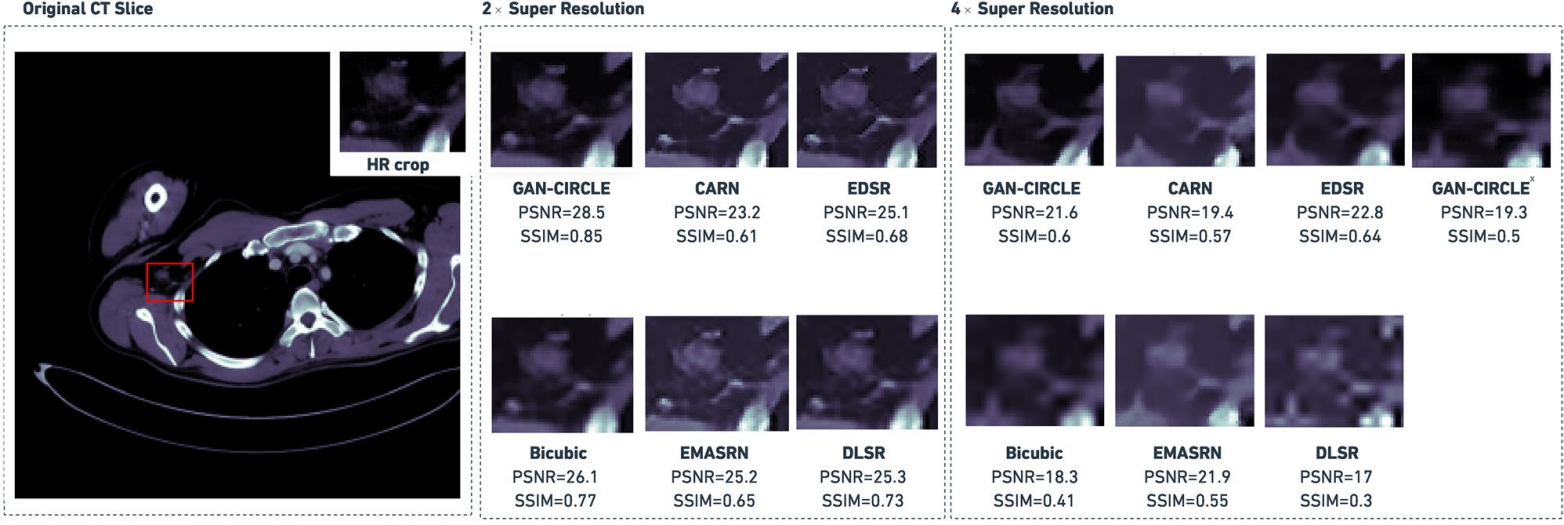
Perceptual quality comparison, on the DeepLesion test images $2\times$ and $4\times$ super-resolved held out for performance evaluation, obtained by the investigated SR methods. The PSNR and SSIM values are shown at the bottom of each super-resolved image. In the case of $4\times$ SR, GAN-CIRCLE^x^ denotes the sequential application of two GAN-CIRCLE instances at $2\times$ SR.

**Figure 4:**
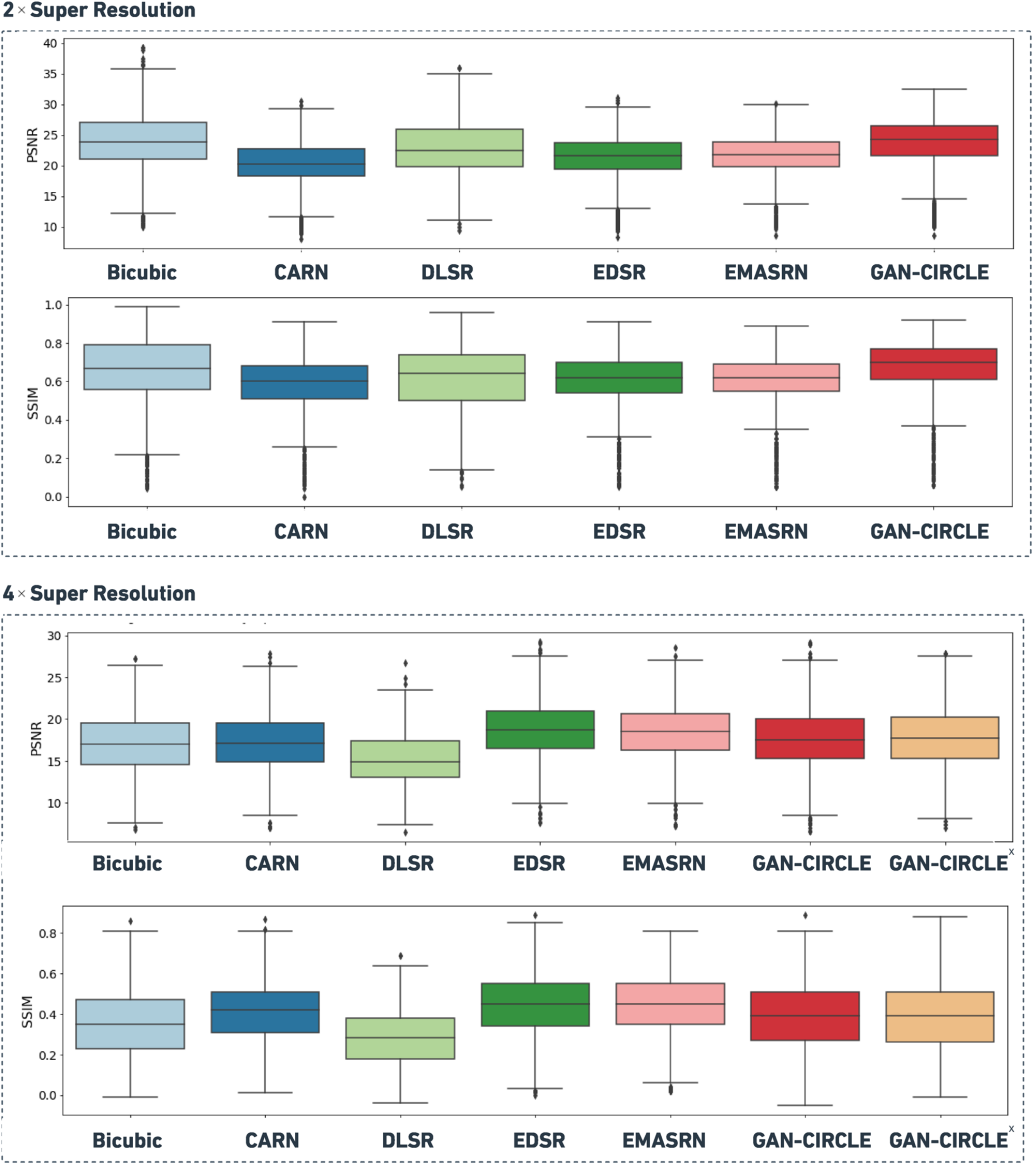
Boxplots comparing PSNR and SSIM metrics for 1000 CT images held out for performance evaluation, super-resolved at $2\times$ and $4\times$ by using the investigated SR methods. In the case of $4\times$ SR, GAN-CIRCLE^x^ denotes the sequential application of two GAN-CIRCLE instances at $2\times$ SR.

Figure 3 shows an example of both $2\times$ and $4\times$ super-resolved images obtained by the considered methods. This example provides a qualitative visual assessment of the super-resolved images. Figure 4 reports the boxplots of the PSNR/SSIM metrics for 1, 000 CT images. From the analysis of Fig. 4, one can see that, at $2\times$ SR, the proposed GAN-CIRCLE-based method achieved higher median values than the other competitors for both the considered metrics (i.e., PSNR and SSIM). On the other hand, at $4\times$ SR, the best SSIM and PSNR values were obtained with the EDSR and EMASRN SR methods. To assess the statistical significance of these results, we performed a Mann–Whitney test for pairwise comparisons (using $\alpha =0.05$). The *p*-values were adjusted *via* the Benjamini–Hochberg method for multiple comparisons. Based on the *p*-values yielded by the statistical test, at $2\times$ SR, GAN-CIRCLE achieved significantly higher PSNR and SSIM values than the other competitors. The only exception is represented by the Bicubic interpolation for which the differences of the median SSIM and PSNR values were not statistically significant. At $4\times$ SR, GAN-CIRCLE showed statistically significant differences, in terms of SSIM and PSNR, when compared against the Bicubic interpolation method and DLSR. The differences were not statistically significant when we compared GAN-CIRCLE against EDSR, EMASRN, and CARN. Finally, at $4\times$ SR, GAN-CIRCLE^x^ produced results comparable to the ones achieved with GAN-CIRCLE.

**Figure 5:**
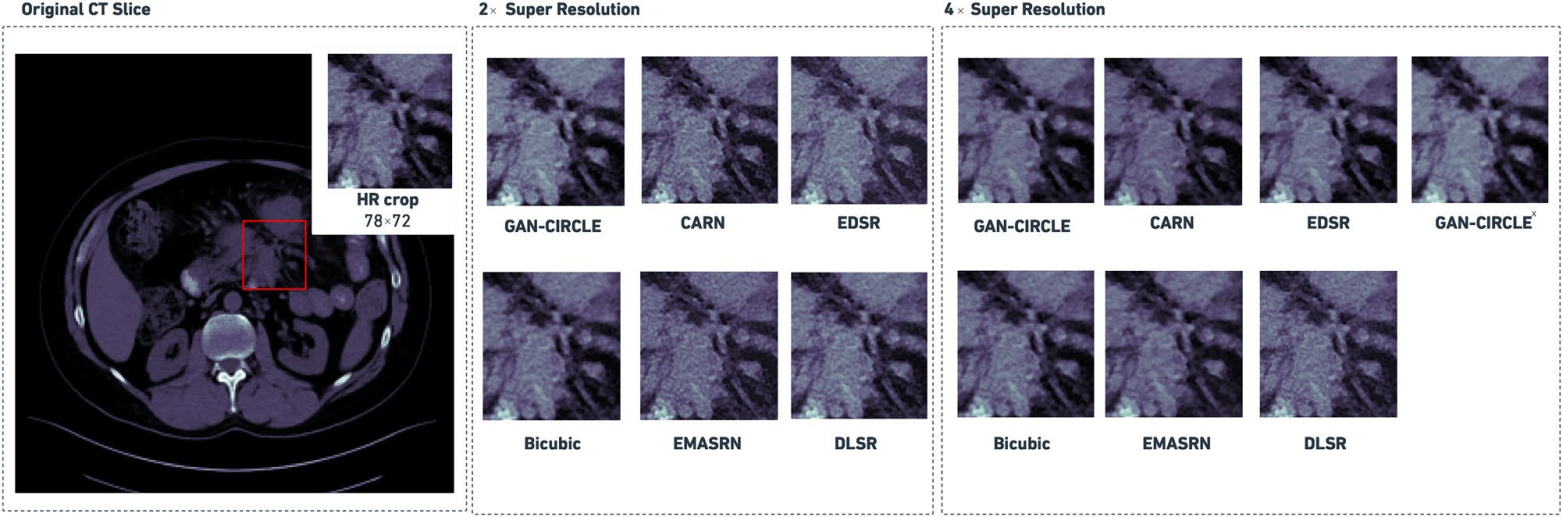
SR example ($2\times$ and $4\times$ factor) using the investigated SR methods from a sample slice randomly selected from the Deeplesion dataset (held-out set). In the case of $4\times$ SR, GAN-CIRCLE^x^ denotes the sequential application of two GAN-CIRCLE instances at $2\times$ SR.

Figure 5 shows a randomly selected example from the Deeplesion dataset to endorse the quality of the produced images and assess the generalization ability of the investigated SR methods. Although PSNR/SSIM are widely adopted evaluation metrics, some studies^19,45^ have demonstrated their limitations on medical image SR tasks since images with low perceptual quality could exhibit high PSNR/SSIM values. Overall, at both $2\times$ and $4\times$ SR, the GAN-generated images were less blurry, with better texture, sharper edges, and visually more similar to the ground truth, as shown in Figs. 3 and 5.

In the downstream radiomic analyses, we focused our attention on the Original images, the super-resolved images *via* the proposed GAN-SR framework (based on SPP and GAN-CIRCLE), and the Bicubic interpolation method. The Bicubic interpolation method obtained, at $2\times$ SR, the best performance (i.e., in terms of PSNR and SSIM) among the considered SR techniques. Moreover, it is commonly available and used in medical image processing.

### Results of the robustness analysis

In this section, we describe and discuss the results of the robustness analysis related to the textural features (in terms of ICC) according to different image groups (i.e., Original, Bicubic, and GAN-SR). Table 1 reports the features with excellent robustness for the considered methods. According to these values, one can observe that all the techniques taken into account produced ten features with excellent robustness. Interestingly, our GAN-SR method shows superior performance in terms of ICC for four features. Moreover, the GAN-SR technique, as well as the Bicubic interpolation, achieved moderate to good robustness for GLRLM LongRunLowGrayLevelEmphasis and GLDM DependenceEntropy, while the features extracted from the Original images resulted in excellent robustness.

**Table 1:** Features that obtained an excellent robustness for at least of the Original, Cubic and GAN-SR image groups.

Feature ID	Feature name	Original	Bicubic	GAN-SR
#1	GLCM Correlation	0.980	0.979	0.984
#2	GLCM DifferenceEntropy	0.846	0.911	0.910
#3	GLCM IDMN	0.996	0.996	0.997
#4	GLCM ID	0.997	0.995	0.998
#5	GLCM MCC	0.633	0.938	0.923
#6	GLCM SumEntropy	0.822	0.897	0.905
#7	GLRLM LongRunLowGrayLevelEmphasis	0.926	0.560	0.631
#8	GLRLM LowGrayLevelRunEmphasis	0.967	0.952	0.944
#9	GLRLM ShortRunLowGrayLevelEmphasis	0.97	0.973	0.925
#10	GLDM DependenceEntropy	0.910	0.870	0.895
#11	GLDM LargeDependenceLowGrayLevelEmphasis	0.985	0.976	0.890
#12	GLDM LowGrayLevelEmphasis	0.986	0.986	0.950
#13	GLDM SmallDependenceLowGrayLevelEmphasis	0.902	0.955	0.946

Table 2 reports the most important features according to the implemented PCA-based procedure. These four features are related to the GLCM matrix (the GLCM characterizes the texture of an image by calculating the occurrences of voxel pairs with specific values in a defined spatial relationship^36^) and, in particular, are the following: Correlation, IDMN, IDN, SumEntropy (Feature IDs: #1, #3, #4, #6). Of particular interest is the SumEntropy feature, defined as the sum of neighborhood intensity value differences, which showed excellent robustness with the GAN-SR method, while it showed good robustness in Original and Bicubic.

Table 2 shows the relative difference (in terms of ICC) on the most important radiomic features between GAN-SR and the Original/Bicubic versions. With reference to the most important features, the GLCM Correlation denotes the linear dependency of gray-level values to their respective voxels in the GLCM; the Inverse Difference Moment Normalized (IDMN) is a measure of the local homogeneity of an image that normalizes the square of the difference between neighboring intensity values by dividing over the square of the total number of discrete intensity values; the Inverse Difference Normalized (IDN) is another measure of the local homogeneity of an image that normalizes the difference between the neighboring intensity values by dividing over the total number of discrete intensity values.

**Table 2:** Relative difference (in terms of ICC) of the GAN-SR against the Original and Bicubic versions on the most important radiomic features according to PCA analysis.

Feature name	Original	Bicubic	GAN-SR	GAN-SR *vs.* Original (%)	GAN-SR *vs.* Bicubic (%)
GLCM Correlation	0.980	0.979	0.984	0.41	0.51
GLCM IDMN	0.996	0.996	0.997	0.1	0.1
GLCM IDN	0.997	0.995	0.998	0.1	0.3
GLCM SumEntropy	0.822	0.897	0.905	10.1	0.89

According to the procedure designed for robustness in the radiomic feature, the optimal binning was found with 64 bins after the perturbation process.

**Figure 6:**
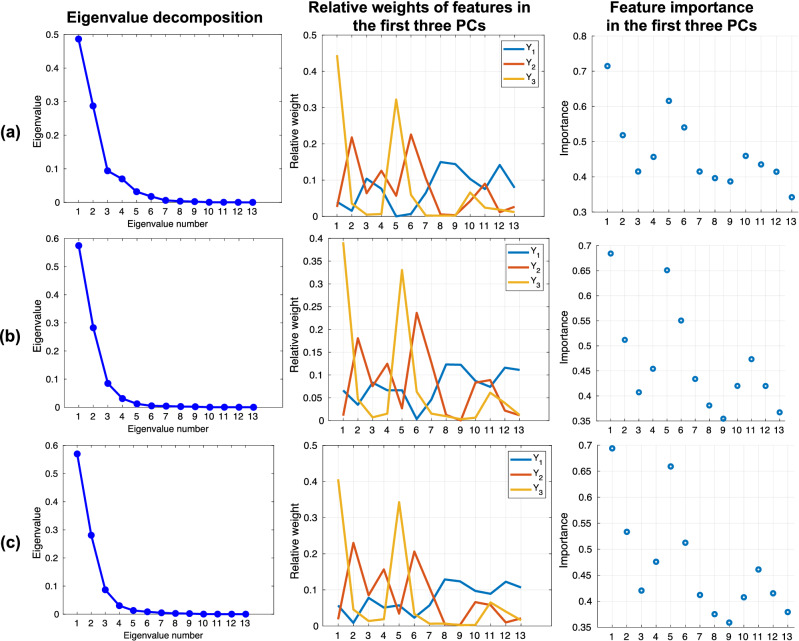
PCA-based analysis of the importance of radiomic features for all image types: (**a**) Original; (**b**) Cubic; (**c**) GAN-SR. The first column shows the line plots of the values of the eigenvalues as a function of the number of eigenvalues. This is useful for the evaluation of the PCs required. The second column shows the relative weights of the original features on the first of three PCs, while the third column depicts the relative importance of the features (according to the IDs defined in Table 1) in the first three PCs.

In Fig. 6, the plots in the left column justify the use of the first three PCs, as the first three eigenvalues cover at least $85\%$ of the trace of the covariance matrix in each group. The plots in the second column show the weights of the original features on the first three PCs, while the third column shows the relative importance of the features in the first three PCs. The most important features (in descending order), for the three groups of images, were as follows:

- Original: #1, #5, #6, #2, #10;
- Bicubic: #1, #5, #6, #2, #11;
- GAN-SR: #1, #5, #2, #6, #4.

Intriguingly, the features with a lower ICC in the GAN-SR method were those of less importance in terms of the PCA. Our GAN-SR method, therefore, increased the robustness of the most important features, compared to the Original and Cubic groups. These highly robust features are expected to generalize well on other and unseen imaging datasets.

## Discussion

This paper presented the first application of GAN-based image SR to radiomic studies. As a proof-of-concept, CT images were considered. In particular, the DeepLesion^23^ dataset was used for training and testing the GAN-SR performance in terms of PSNR and SSIM. The performance of the proposed method was compared against recent state-of-the-art methods for image SR. To quantitatively assess the performance of the proposed framework and compared it against the considered state-of-the-art SR techniques, we relied on two commonly used metrics: PSNR and SSIM. Moreover, to carefully assess the performance of the proposed GAN-CIRCLE-based SR method at $4\times$ SR, we compared the native $4\times$ GAN-CIRCLE SR against the sequential application of two GAN-CIRCLE instances at $2\times$ SR (i.e., GAN-CIRCLE^x^). Experimental results showed that, at $2\times$ SR, the proposed GAN-CIRCLE-based method achieved better performance (with statistical significance, except for the Bicubic interpolation) than the other competitors for both the considered metrics. On the other hand, at $4\times$ SR, the best SSIM and PSNR values were obtained with the EDSR and EMASRN SR methods. Still, the performance of the proposed framework was comparable (i.e., no statistically significant difference) to the two best performers. According to the results achieved, we can state that the proposed SR framework can obtain competitive performance with respect to the considered competitors across the tested SR factors. Additionally, the visual assessment of the super-resolved images showed that, in general, the GAN-CIRCLE-based method produced images with better texture and sharper edges, and they looked visually more similar to the ground truth HRCT.

The experimental evidence allowed us to choose the proposed GAN-CIRCLE framework, integrating the SPP, as the most suitable approach for evaluating the impact of advanced image SR methods in oncological imaging. Therefore, the resulting GAN-SR model was leveraged to assess the robustness of the radiomic features extracted from the images of the TCIA NSCLC CT dataset^46^. This assessment required the computation of the ICC to identify the most robust features against the variations of the number of bins used in the quantization step. The ICC values, calculated for three different image groups (i.e., Original, Bicubic, and GAN-SR), showed that all the techniques obtained ten texture features with excellent robustness. Still, the proposed GAN-SR method presented superior ICC values in four of the ten features with excellent robustness. Finally, a PCA was performed to identify the relative importance of the radiomic features in the proposed GAN-SR technique. The results obtained from this analysis are particularly interesting as the features with the lowest ICC values are the ones deemed less relevant in terms of the PCA analysis. On the contrary, GAN-SR increased the robustness of the most important features compared to the Original and Bicubic groups. The result is relevant because the highly robust features identified by GAN-SR might generalize well on other CT datasets. The results of this study could pave the way for the application of GAN-based image SR techniques for studies of radiomics for robust biomarker discovery^47,48^.

Along with the novelties in lesion-focused GAN-based SR, this work belongs to the research strand dedicated to the analysis of robustness in radiomic features, with particular interest in oncological imaging. As a matter of fact, the investigation techniques used in our study were consistent with the state-of-the-art: the ICC was adopted in radiomic feature robustness analyses that assessed the impact of different imaging acquisition and reconstruction parameters^6,7,49^, as well as image perturbations^4,5,8^. Moreover, we identified the most important features in an agnostic manner, which is independent on a particular classification/prediction task at hand, by using a PCA-based investigation^43^.

The main limitation of the proposed SR method is inherent to its lesion-focused approach, which relies on a lesion detection step for ROI identification that limits the application of this method to datasets with a pre-existing mapping of ROIs. Regarding this matter, our methodological approach could be extended to include a lesion detection task as in^19^, to allow for CT images without lesion annotations in the training process. Considering that our GAN-SR method currently performs only in-plane 2D image SR, to avoid the effect of slice thickness variability^6,7^, GAN-based SR along the *z*-axis (i.e., yielding thinner slices) might relieve the problem related to highly anisotropic voxels^50,51^. Moreover, since our GAN-SR model does not remarkably improve PSNR/SSIM values, we could conduct feature recalibration, such as *via* self-attention mechanisms, to obtain features more similar to the ones of the original images^21,52–54^. Concerning future radiomics applications, since we showed the results on a homogeneous subset of the NSCLC-Radiomics dataset, we plan to test the generalization ability of GAN-extracted radiomic features on the whole dataset, considering variations on CT image acquisition and reconstruction parameters. In particular, a classification/prediction modeling task for NSCLC staging and type would be beneficial^24^.
